# Promoting work ability in a structured national rehabilitation program in patients with musculoskeletal disorders: outcomes and predictors in a prospective cohort study

**DOI:** 10.1186/1471-2474-14-57

**Published:** 2013-02-06

**Authors:** Kjerstin G E Stigmar, Ingemar F Petersson, Anna Jöud, Birgitta E M Grahn

**Affiliations:** 1 Kommunhälsan Occupational Health Services, Box 1222, Växjö S 351 12, Sweden; 2 Department of Health Sciences, Division of Physiotherapy, Lund University, Lund, Sweden; 3 Research and Development Kronoberg, Box 1223, Växjö, S 351 12, Sweden; 4 Department of Orthopedics, Clinical Sciences Lund, Lund University, WHO Collaborating Centre for Evidence-Based Healthcare in Musculoskeletal Disorders, Lund, Sweden; 5 EPI-centrum, County Council of Skåne, Lund, Sweden; 6 FoU-Kronoberg, Box 1223, Växjö, S 351 12, Sweden

**Keywords:** Sick leave, Musculoskeletal pain, Multimodal rehabilitation, Health related quality of life, Function

## Abstract

**Background:**

Musculoskeletal disorders (MSDs) are a major reason for impaired work productivity and sick leave. In 2009, a national rehabilitation program was introduced in Sweden to promote work ability, and patients with MSDs were offered multimodal rehabilitation. The aim of this study was to analyse the effect of this program on health related quality of life, function, sick leave and work ability.

**Methods:**

We conducted a prospective, observational cohort study including 406 patients with MSDs attending multimodal rehabilitation. Changes over time and differences between groups were analysed concerning function, health related quality of life, work ability and sick leave. Regression analyses were used to study the outcome variables health related quality of life (measured with EQ-5D), and sick leave.

**Results:**

Functional ability and health related quality of life improved after rehabilitation. Patients with no sick leave/disability pension the year before rehabilitation, improved health related quality of life more than patients with sick leave/disability pension the year before rehabilitation (*p =* 0.044). During a period of −/+ four months from rehabilitation start, patients with EQ-5D ≥ 0.5 at rehabilitation start, reduced their net sick leave days with 0.5 days and patients with EQ-5D <0.5 at rehabilitation start, increased net sick leave days with 1.5 days (*p =* 0.019). Factors negatively associated with sick leave at follow-up were earlier episodes of sick leave/disability pension, problems with exercise tolerance functions and mobility after rehabilitation. Higher age was associated with not being on sick leave at follow-up and reaching an EQ-5D ≥ 0.5 at follow-up. Severe pain after rehabilitation, problems with exercise tolerance functions, born outside of Sweden and full-time sick leave/disability pension the year before rehabilitation were all associated with an EQ-5D level < 0.5 at follow-up.

**Conclusions:**

Patients with MSDs participating in a national work promoting rehabilitation program significantly improved their health related quality of life and functional ability, especially those with no sick leave. This shows that vocational rehabilitation programs in a primary health care setting are effective. The findings of this study can also be valuable for more appropriate patient selection for rehabilitation programs for MSDs.

## Background

In western countries, musculoskeletal disorders (MSD) are a major reason for work ability limitations and sick leave [1-3] and causes about one third of the total amount certified sick leave [4]. Musculoskeletal disorders are affected by psychosocial factors; both at start and in the disease course [5,6].

In the late 90s and turn of the century, there was a steady increase in the amount of people sick-listed in Sweden. The same trend was seen also in Norway and the Netherlands [7]. To break the trend, different changes in the systems have been made [8-12] and major resources been transferred from the social insurance system to the health care system, as incentives to improve rehabilitation and sick leave management. From 2009, approximately one hundred million Euros have been available on a yearly basis for the regional health authorities to improve the sick-listing process [13].

A structured rehabilitation time-schedule was introduced, which highlighted the employer’s responsibilities and also how work ability should be assessed and improved [11]. Another major reform was the introduction of a structured, national rehabilitation program, aiming at promoting work ability, by offering early, evidence-based rehabilitation directed to and ensured for patients with mild to moderate mental disorders and persons with MSD, mainly neck, shoulder and back pain [10]. For patients with MSD, multimodal rehabilitation (MMR) was offered after referral from primary health care (PHC) system. Different MMR programs have been evaluated during the past years and MMR has been found to be effective on return to work (RTW) [14] and cost-effective [15]. Combining MMR and work place interventions are found effective on RTW [16,17].

The concept of work ability is complex [18-21] and there are also divergent interpretations [22,23]. Different health professionals experience work ability as difficult to assess [22,24-27] and there are no single measure that can capture all factors contributing to work ability [28]. Therefore it is important to evaluate different outcomes related to work ability to better understand the concept. In this study we focus on work ability in a broader perspective. The aim of this study was to study the outcomes of MMR in a national rehabilitation program and associated factors of changes in health related quality of life (HRQoL) and sick leave.

## Method

We conducted an explorative, prospective observational study in a well-defined cohort and linked this to register data on sick leave.

### Setting and procedure

In Sweden, the responsibility for providing health care is decentralised to the county councils. Both public and private health care providers have the same tax-based financing system and apart from a small co-pay the residents are entitled to free health care. Skåne County (Region Skåne) is the southernmost part of Sweden with a population of 1.3 million inhabitants, which correspond to 1/8 of the total Swedish population and is representative for the whole Swedish population, in terms of demographics. The Skåne Health Care Register (SHCR) contains routinely collected data of all health care in the region. All data is on an individual level, coded by personal identification number (PIN), a unique number automatically assigned to all residents. In this study, data collection took place in a natural setting at different PHC centres in the county; both private and public. Patients with MSD, seeking PHC were offered MMR, through the national rehabilitation program and were followed with self-administered questionnaires at MMR start and after finished MMR. A three month follow-up was also conducted. A questionnaire, completed by health care professionals, was answered at MMR start and after completed MMR. Also basic demographic data was collected and each patient consultation was registered with date, type of treatment and profession. The parameters were consecutively reported from different health care providers. These registrations were linked to economical compensations for the PHC centres and also for the purpose of provide follow-ups for the responsible authority and to the particular health care unit.

**Figure 1:**
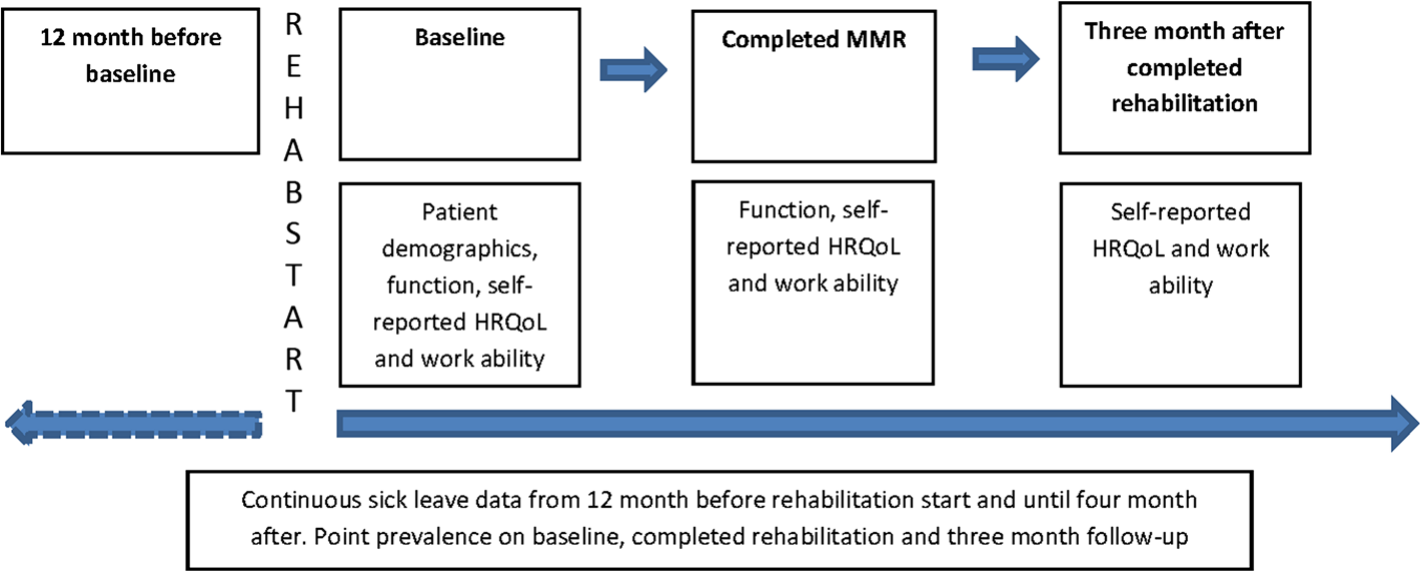
Flowchart of outcomes.

In Sweden, sick leave insurances include the whole working population from the age of 16 or older, and are tax-financed from day 15. Sick leave data was obtained from the Swedish Social Insurance Agency (SSIA) (Figure 1).

### Patients

**Figure 2:**
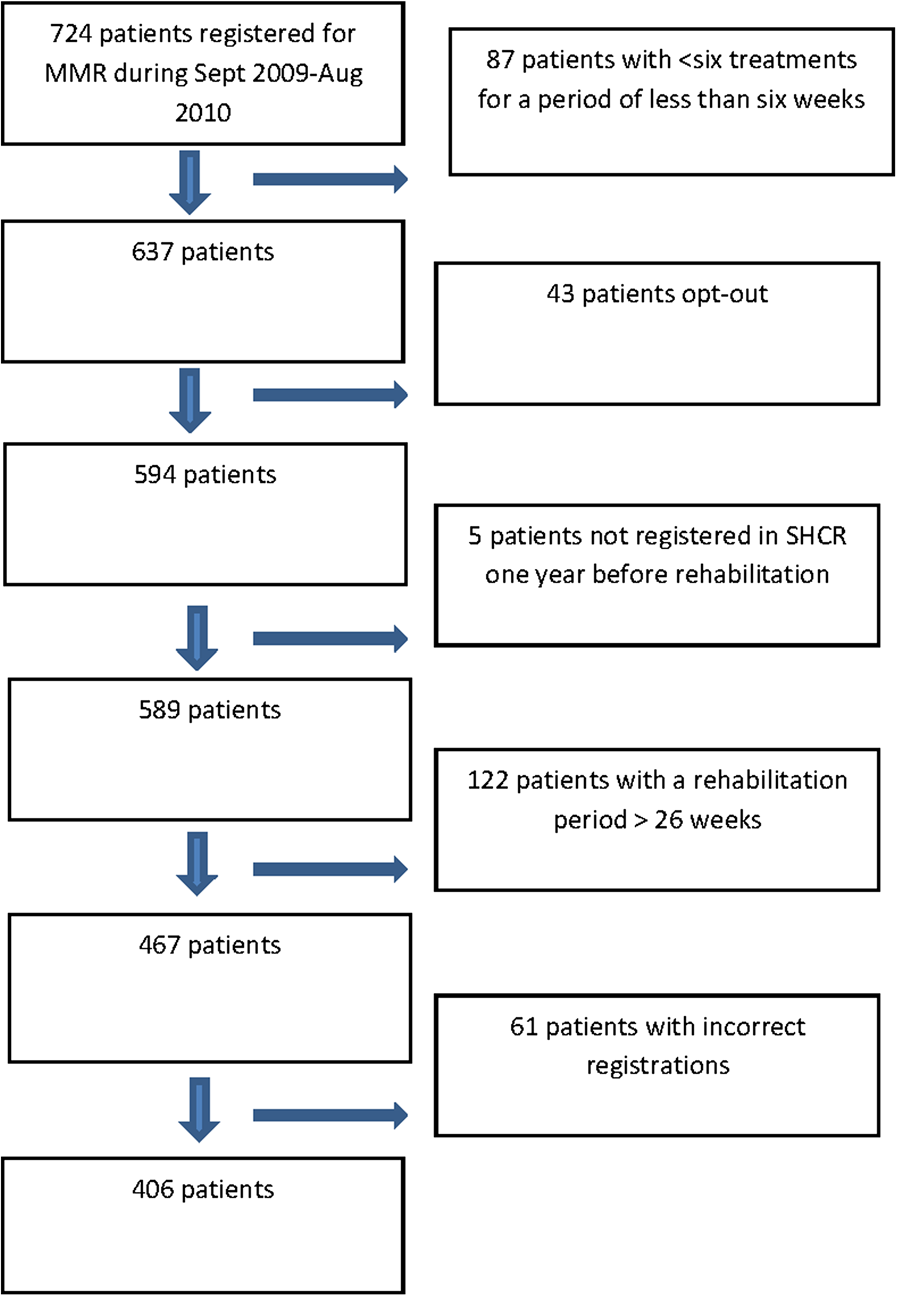
Flowchart on inclusion and exclusion in the cohort.

The setting of patients was a pre-defined cohort of all patients that had been consecutively notified for MMR in Skåne County, during September 2009 and until August 2010. The cohort included 724 patients with MSD. This is the first cohort of patients that had access to MMR through the national rehabilitation program [10]. In line with the intention of the intervention, patients receiving at least six treatments for a period of at least six weeks were included, which resulted in 637 patients. To be able to link sick leave data to patient data, all patients were sent a letter of consent with opt-out opportunity. In total 43 patients decided not to participate. Sick leave data from SSIA were linked for the remaining 594 patients. Five patients not registered in Region Skåne, one year before MMR start, were excluded. To stay close to the intention of the National rehabilitation program, patients that had a rehabilitation period lasting for more than 26 weeks were excluded and also those patients that had invalid time series for date registrations. The final study cohort consisted of 406 patients (Figure 2).

### Interventions

MMR comprised of evidence-based assessments and rehabilitation interventions [10]. At least three different professionals must be included in the team, whereof one had to be a physician. The interventions must last for minimum six weeks, two-three times a week and should be coordinated.

### Outcomes

#### Self-reported health related quality of life (HRQoL)

The self-administered, generic questionnaire EQ-5D was used [29,30] and in this study the five-question part was analysed. Each question has three answering alternatives; 1–3, and 1 correspond to no problems. The answers were merged into one total EQ-5D from −0.59 -1, according to the United Kingdom (UK) tariff (since no tariff for Sweden is available) and 1 correspond to full HRQoL [31-33].

#### Self-reported work ability

All patients that had an employment or were self-employed answered a question if they perceived work ability limitations the last week, while at work and due to pain (yes/no).

#### Function

The health care professionals answered questions about their evaluation of the patient’s functional limitations, grounded on International Classification of Functioning, Disability and Health (ICF) [34]. These questions graded sensation of pain (b280), problems with exercise tolerance functions (b455) and mobility of joint functions (b710). The answering alternatives were graded in five steps, from no limitation (0) to total limitation (4).

#### Sick leave data

Sick leave data for a period of 12 month before MMR start and four month after MMR start were collected from the register at SSIA. This data were reported in different types of compensations and reported as disability pension (DP), total sick leave (SL), which includes sick leave in prevention, work related injury compensation and rehabilitation compensation. Total burden of sick compensations (SLDP) was also reported. SLDP may comprise both SL and DP or only one of the compensations. SL and DP were reported in net days (net days computed as full working days of sick leave compensation, i.e., two half days of SL (or DP) correspond to one full net day) for each month, on one-year basis and also as period- and point prevalence. New episodes on SL or DP were also reported.

### Analysis

#### Grouping and organisation of data

When describing characteristics of the study population, comparing functions and EQ-5D, the cohort was divided in three different groups, based on the total burden of net days SLDP the year before MMR; no SLDP, part-time SLDP (< 360 days) and full-time SLDP (≥360 days). When comparing SL, the group was divided in having an EQ-5D < or ≥ 0.5 at MMR start. Diagnoses were registered according to the Swedish translation of International Classification of Diseases and Related Health Problems (ICD) 10 system [35] and grouped together and presented in four different groups; myalgia or pain (M791, M799P, R52) neck-shoulder pain (M42, M530, M531, M50-, M750, M751, M754, M759P, T918A,), back pain (M51, M533, M543, M544, M545, M546, M549P) and rheumatism UNS (M790) (Table 1).

**Table 1:** Baseline characteristics of the study population (n = 406)

	**No**				**Part-time**				**Full-time**			
	***n =*** **123**				***n =*** **214**				***n =*** **69**			
	***n***	**%**	**Mean**	**SD**	***n***	**%**	**Mean**	**SD**	***n***	**%**	**Mean**	**SD**
Age			42.51	10.15			45.76	9.82			47.71	8.22
Women/men	94/29	76.4/23.6			174/40	81.3/18.7			56/13	81.2/18.8		
Born in Sweden y/n	102/21	82.9/17.1			165/49	77.1/22.9			43/26	62.3/37.7		
Married y/n	65/68	52.8/47.2			120/94	56.1/43.9			35/34	50.7/49.3		
Children home y/n	73/50	59.3/40.7			117/97	54.7/45.3			37/32	53.6/46.4		
Empl/self-empl y/n	84/39	68.3/31.7			148/66	69.2/30.8			17/52	24.6/75.4		
**Education**												
No/elementary school	15	12.2			45	21.0			20	29.0		
Upper sec school	66	53.7			126	58.9			39	56.5		
University	42	34.1			43	20.1			10	14.5		
**Diagnos**												
Myalgia and pain	62	50.4			90	42.0			39	56.6		
Neck-shoulder pain	26	21.1			47	22.0			12	17.4		
Back pain	31	25.2			61	28.5			13	18.8		
Rheumatism UNS	4	3.3			16	7.5			5	7.2		

Before regression analyses, variables with several different answering alternatives were grouped together so there were no more than three different alternatives for each variable. The first level served as reference value (1). The continuous variable age, were dichotomised in < 40 years respectively ≥ 40 years and EQ-5D into < 0.5 or ≥ 0.5. In the regression analyses of sick leave at month four after MMR start, patients with full-time DP the year before MMR (*n =* 45) were excluded from the cohort. In the variable SLDP episodes the year before MMR start, patients with part-time DP were counted as having one episode.

#### Statistical analyses

Descriptive statistics were presented by their frequencies and significances were evaluated by the Chi-Square test and independent sample t-test, when comparing means. Paired sample t-test was used when analysing mean differences over time. The Mann–Whitney Test was used when comparing ordinal data over time and between group differences. Changes in functions were merged into improved, not improved and impaired, before comparing groups. Independent sample t-test and paired sampled t-test were used to compare differences within and between groups. To find out about associated factors for sick leave at month four after MMR start and EQ-5D < or ≥ 0.5 at follow-up, regression analyses were performed. The crude model was comprised of univariate regression analyses for each variable. Model I was comprised of variables on function and HRQoL at MMR start and completed MMR, and backward regression analysis was performed. In model II, adjustments were made for background characteristics. The results are presented in odds ratio (OR), confidence interval 95% (CI) and also Nagelkerke, R^2^, for model precision of outcome. Significant *p-*values (< 0.05) are marked in bold face [36,37]. Data were analysed with IBM Statistical software Package for the Social Sciences (SPSS), version 20.0.

### Ethics

This study has been approved by the Regional Ethical Review Board in Lund Dnr 2011/169 and approved by Skåne County health care authorities was also received.

## Results

### Baseline demographics

The study cohort (*n =* 406*)* consisted of 20.2% men and 79.8% women, with a mean age of 45.1 (SD 9.83) (Table 1). Patients with no SLDP the year before MMR, were slightly younger than patients with part-time SLDP (*p =* 0.004) and patients with full-time SLDP (*p <* 0.000). The group with no SLDP had higher education level compared to the group with part-time SLDP (*p =* 0.007) and the group with full-time SLDP (*p <* 0.001). Patients with full-time SLDP the year before MMR were to a greater extent not employed/self-employed (75.4%), than both the other groups (*p <* 0.000). These patients were also to a higher extent born outside Sweden compared to no SLDP (*p =* 0.001) and those with part-time SLDP (*p =* 0.016).

There were no differences in how symptoms were distributed when comparing the three groups based on SLDP, but there were gender differences. Most women had myalgia or pain (49.4%) whilst for men, back pain and myalgia or pain were most common and equally distributed (36.1% each).

At MMR start, 6.4% of the whole group was on sick leave in prevention, as they were part of rehabilitation for purpose of preventing future sick leave or shortening an on-going sick leave spell. 36.7% were on SL, 25.4% had DP and 44.1% had no SLDP. The total burden of SL and DP for each person, one year before MMR, was mean144 net days (SD 141.50), where 73.4 net days (SD 73.37) corresponded to SL and 70.6 (SD 12.47) net days to DP. No gender differences were noted.

The original cohort comprised of 724 patients and 318 patients were excluded from the cohort, (Figure 2) of which 22.6% were men and 77.4% women. Mean age was 45.0 years (SD 10.73), which is in line with the final study cohort. Register data on sick-leave was not available for the pre-defined original cohort.

### Multimodal rehabilitation (MMR)

The patients had a MMR period of mean 94.3 days (SD 38.76) and had mean 17.4 treatments (SD 7.13). The group with no SLDP had just over two visits fewer compared to the group with part-time SLDP (*p =* 0.005) and full-time SLDP (*p =* 0.011). Physicians (40.8%) and physiotherapists (34.4%) were the most common professionals when registering for MMR and also discharging MMR (43.6% resp. 33.5%). Also other health care professionals as psychologists and occupational therapists were involved in MMR but physiotherapists were the most frequently occurring health care professional. Three or more different health care professionals were involved in 62% of the patients’ rehabilitation.

### Health related quality of life

**Figure 3:**
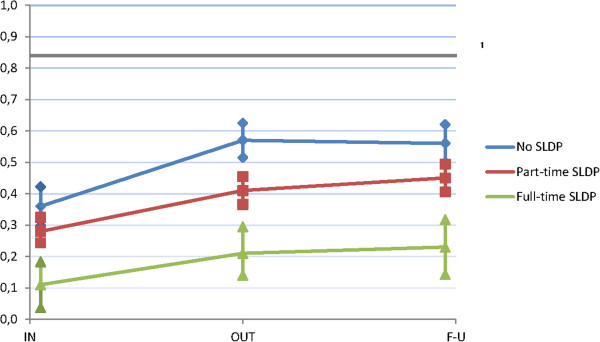
**Health related quality of life (HRQoL) measured by EQ-5D. **Mean HRQoL at multimodal rehabilitation (MMR) start (IN), completed MMR (OUT) and at three month follow-up (FU), in patients with no (*n =* 123), part-time (*n =* 214) or full-time (*n =* 69) sick leave/disability pension (SLDP) the year before MMR. ^1 ^EQ-5D = 0.84, a Swedish normal population in two counties. 95% confidence interval (CI).

EQ-5D at MMR start, after completed MMR and at follow-up differed between the groups with no, part-time and full-time SLDP (Figure 3). There were significant improvements between MMR start and completed MMR within all three groups and for the group with part-time SLDP, there was a slight improvement between completed MMR and follow-up (*p =* 0.035). The group with no SLDP improved more between MMR start and completed MMR compared to the group with part-time SLDP (*p =* 0.044) and also full-time SLDP (*p =* 0.044). No differences in improvement, between the three groups were seen between completed MMR and three month follow-up.

### Function

All functions were assessed as improved between MMR start and completed MMR, for all three groups (Table 2). There were differences in pain at MMR start, between the group with no and full-time SLDP (*p <* 0.000). Also at completed MMR there were differences in pain between no and part-time SLDP (*p* < 0.000) and no and full-time SLDP (*p* < 0.000). There were differences in improvement between the three groups (*p<*0.000).

**Table 2:** **Function at MMR start (IN) and completed MMR (OUT) *n = *406**

	**NO**					**Part-time**					**Full- time**					
	***n = *123**					***n = *214**					***n = *69**					
	**IN**		**OUT**			**IN**		**OUT**			**IN**		**OUT**			
	*n*	%	*n*	%	*p*^*1*^	*n*	*%*	*n*	%	*p*^*2*^	*n*	%	*n*	%	*p*^*3*^	*p*^*4*^
**Pain b280**					<0.000					<0.000					<0.000	
No	0	0	7	5.7		1	0.5	3	1.4		1	1.4	0	0		
Mild	23	18.7	49	39.8		20	9.3	41	19.2		2	2.9	11	15.9		
Moderate	56	45.5	49	39.8		90	42.1	90	42.1		22	31.9	23	33.3		
Severe	43	35.0	18	14.6		102	47.7	77	36.0		42	60.9	33	47.8		
Total	1	0.8	0	0		1	0.5	2	0.9		2	2.9	2	2.9		
Missing value								1	0.5							
Improved	66	53.7				53	24.9				19	27.5				<0.000
Unchanged	48	39.0				151	70.9				44	63.8				
Impaired	9	7.3				9	4.2				6	8.7				
**Exerc tolerance b455**					<0.000					<0.000					<0.000	
No	26	21.1	50	40.7		27	12.6	38	17.8		7	10.1	10	14.5		
Mild	32	26.0	33	26.8		48	22.4	70	32.7		12	17.4	16	23.2		
Moderate	43	35.0	28	22.8		94	43.9	83	38.8		21	30.4	21	30.4		
Severe	20	16.3	11	8.9		40	18.7	18	8.4		25	36.2	19	27.5		
Total	2	1.6	1	0.8		5	2.3	4	1.9		4	5.9	3	4.3		
Missing value								1	0.5							
Improved	51	41.5				70	32.9				19	27.5				0.054
Unchanged	64	52.0				125	53.6				44	63.8				
Impaired	8	6.5				18	8.5				6	8.7				
**Mobility b710**					<0.000					<0.000					<0.000	
No	7	5.7	21	17.1		13	6.1	24	11.2		5	7.2	4	5.8		
Mild	46	37.4	61	49.6		52	24.3	67	31.3		8	11.6	16	23.2		
Moderate	39	31.7	22	17.9		75	35.0	70	32.7		26	37.7	24	34.8		
Severe	20	16.3	14	11.4		47	22.0	39	18.2		21	30.4	17	24.6		
Total	0	0	0	0		1	0.5	0	0		2	2.9	2	2.9		
Missing value	11	8.9	5	4.1		26	12.1	14	6.5		7	10.1	6	8.7		
Improved	41	38.0				49	27.4				12	20.7				0.037
Unchanged	61	56.5				114	63.7				43	74.1				
Impaired	6	5.6				16	8.9				3	5.2				

*p*^*1*^*= Changes in function between rehab start and completed rehab, within the group with no SLDP the year before; p*^*2*^*= Changes in function between rehab start and completed rehab, within the group with part-time SLDP the year before; p*^*3*^*= Changes in function between rehab start and completed rehab, within the group with full-time SLDP the year before; p*^*4*^*= Chi-2 trend. Comparisons in improvement between all three groups.*

Exercise tolerance functions were different when comparing no and full-time SLDP, both at MMR start (*p =* 0.005) and completed MMR (*p* < 0.000). Also when comparing the group with no and part-time SLDP, exercise tolerance functions were different at completed MMR (*p* < 0.000). There were also differences between the group with part-time and full-time SLDP (*p =* 0.001). There were no differences between the three groups, in improvement of exercise tolerance functions (*p=* 0.054)**.**

Mobility of joints functions were different when comparing the group with no SLDP and full-time SLDP at MMR start (*p =* 0.001) and at completed MMR (*p* < 0.000). At MMR start, there were also differences between no and part-time SLDP (*p =* 0.001). There were differences in improvement between the three groups (*p=* 0.037).

### Sick leave and disability compensations

**Figure 4:**
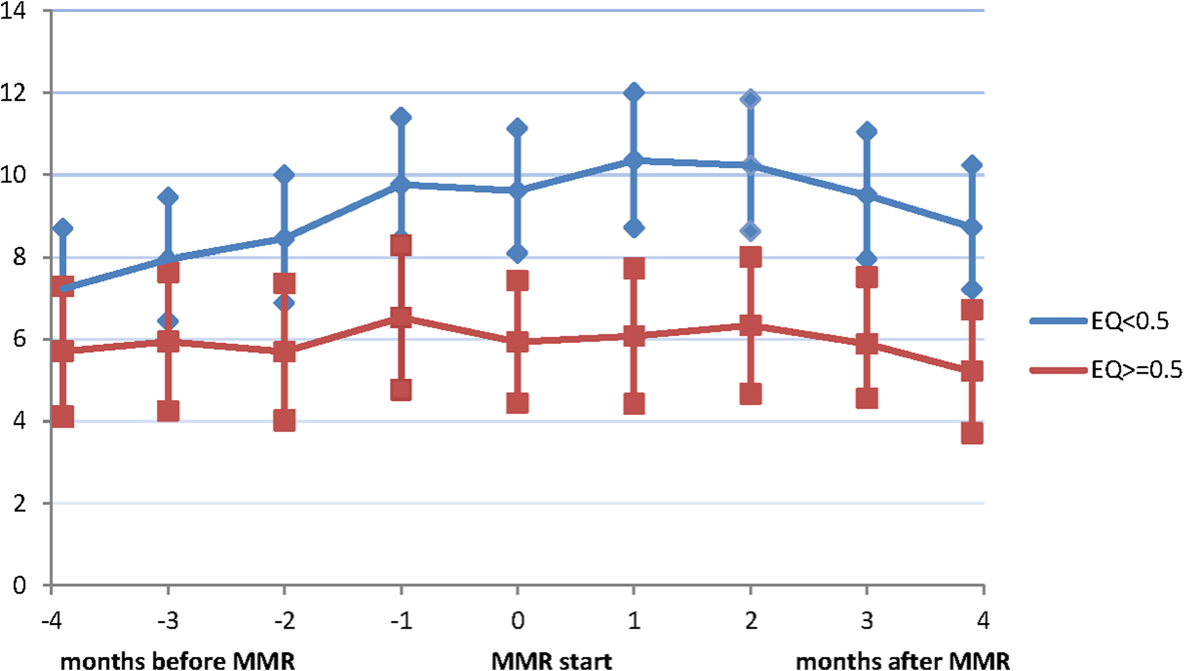
**Net sick leave days four month before until four month after multimodal rehabilitation (MMR)start. **Mean net sick leave days four month before until four month after MMR start in patients with EQ-5D < 0.5 (*n =* 253 ) respectively ≥0.5 (*n =* 153 ) at MMR start. 95% confidence interval (CI).

Comparisons were made between patients with EQ-5D < 0.5 respectively ≥ 0.5, at MMR start. There were no differences in net SL days four months before MMR start, but the group with EQ-5D < 0.5 had more net SL days at +/− two weeks from MMR start t (*p =* 0.002) and four month after MMR start (*p =* 0.003). Also, there were differences between the groups, in how net SL days developed for the period month four before MMR until MMR start (*p =* 0.037) and also for the whole period (*p =* 0.057*)* (Figure 4). In the group with EQ-5D ≥ 0.5, net SL days decreased with mean 0.5 days (SD10.28) under the period from month four before MMR start until four month after MMR start (*p =* 0.552) and in the group with EQ-5D < 0.5 net SL days increased with mean 1.5 days (SD 10.08 ) (*p =* 0.019).

### Work ability

**Figure 5:**
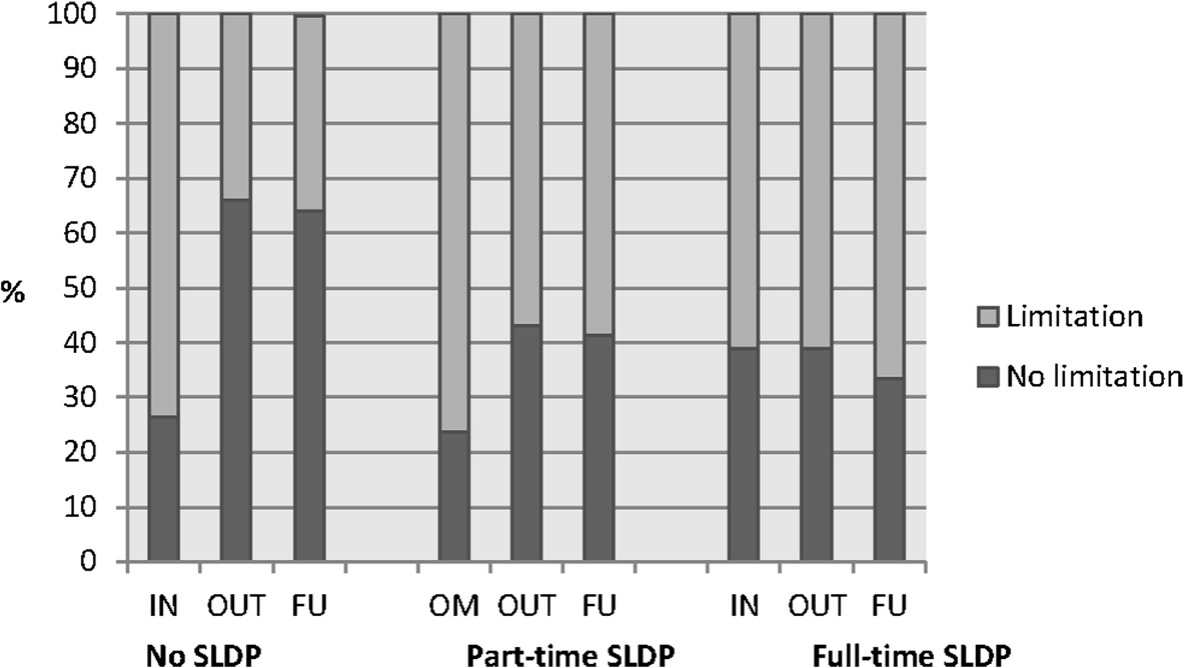
**Self-reported work ability at multimodal rehabilitation (MMR) start, completed MMR and at three month follow-up. **Self-reported work ability (limited or not limited) the last week, while at work and due to pain, at multimodal rehabilitation (MMR)start (IN), completed MMR (OUT) and follow up (FU) in employed/self-employed patients with no, part-time or full-time sick leave/disability pension (SLDP) the year before MMR. No SLDP: *n =* 87 (IN), *n =* 88 (OUT), *n =* 93 (FU); Part-time SLDP: *n =* 151 (IN), *n =* 144 (OUT), *n =* 140 (FU); Full-time SLDP: *n =* 18 (IN), *n =* 18 (OUT), *n =* 15 (FU).

In all three groups (no, part-time or full-time SLDP) patients reported work ability limitations at MMR start, 73.6, 76.2 and 61.1% respectively (Figure 5). When comparing the three groups, there were no significant differences in work ability limitations at MMR start. After completed MMR, there were differences between the groups. In the group with no SLDP, 34.1% reported work ability limitations, compared to 56.9% in the group with part-time SLDP (*p =* 0.001) and 61.1% in the group with full-time SLDP (*p =* 0.032). When comparing the group with part-time and full-time SLDP, no differences were seen at completed MMR (*p =* 0.736). At follow-up, 35.5% in the group with no SLDP reported work ability limitations, compared to 58.6% of those with part-time SLDP (*p =*0.001) and 66.7% of those with full-time SLDP (*p =* 0.022), but no differences were seen between part-time and full-time SLDP (*p =*0.544).

### Factors associated with EQ-5D < or ≥ 0.5 at three month follow-up

In the crude model, functional limitations at MMR start and completed MMR were associated with EQ-5D < 0.5. Full SLDP the year before MMR and background factors, such as not being born in Sweden and being un-employed was also associated with EQ-5D < 0.5. University education was significantly associated with EQ-5D ≥ 0.5.

In model I (R^2^ = 0.19) more severe sensation of pain at completed MMR, moderate and more severe problems with exercise tolerance functions at MMR start were significantly associated with having an EQ-5D < 0.5 at three month follow up.

In the final, adjusted model II (R^2^ = 0.28) more severe sensation of pain at completed MMR (OR 0.2, CI 0.1–0.5, *p <* 0.000), moderate (OR 0.5, CI 0.3–0.9, *p =* 0.032) and more severe (OR 0.4, CI 0.2–0.8, *p =* 0.012) problems with exercise tolerance functions, being on full-time SLDP the year before MMR (OR 0.4, CI 0.2–0.9, *p =* 0.027), not being born in Sweden (OR 0.4, CI 0.2–0.6, *p <* 0.000) were significantly associated with an EQ-5D < 0.5 at three month follow-up. Being older than 40 years (OR 1.8, CI 1.1–3.1, *p =* 0.026) was significantly associated with having an EQ-5D ≥ 0.5 at three month follow-up (Table 3).

**Table 3:** Regression models for EQ–5D ≥0.5 at three months follow–up

	**Crude Model**		**Model I**		**Model II**	
			**R**^**2**^ **= 0.192**		**R**^**2**^ **= 0.279**	
	**OR**	**CI**	**OR**	**CI**	**OR**	**CI**
Sensation of pain (b280) IN	1					
Moderate	0.451	0.189–1.076				
Severe/total	**0.123**	**0.503–0.290**				
Sensation of pain (b280) OUT	1		1		1	
Moderate	**0.430**	**0.242–0.763**	0.632	0.337–1.183	0.678	0.350–1.314
Severe/total	**0.117**	**0.064–0.212**	**0.219**	**0.114–0.423**	**0.232**	**0.113–0.474**
Problems with exercise tolerance functions (b355) IN	1		1		1	
Moderate	**0.515**	**0.319–0.830**	**0.574**	**0.330–0.998**	**0.529**	**0.296–0.847**
Severe/total	**0.232**	**0.134–0.400**	**0.359**	**0.189–0.682**	**0.417**	**0.211–0.822**
Problems with exercise tolerance functions (b355) OUT	1					
Moderate	**0.536**	**0.343–0.839**				
Severe/total	**0.195**	**0.103–0.369**				
Problems with mobility in joints function (b710) IN	1					
Moderate	0.752	0.456–1.240				
Severe/total	**0.404**	**0.233–0.702**				
Problems with mobility in joints function (b710) OUT	1					
Moderate	**0.595**	**0.369–0.960**				
Severe/total	**0.255**	**0.144–0.451**				
Sex	1					
Sex (man)	0.805	0.494–1.311				
SLDP–year before (no)	1				1	
Part–time	0.733	0.459–1.171			1.216	0.681–2.174
Full–time	**0.264**	**0.142–0.491**			**0.419**	**0.194–0.907**
Married (yes)	1					
no	1.134	0.762–1.687				
Born in Sweden	1				1	
Not	**0.277**	**0.171–0.447**			**0.372**	**0.214–0.645**
Age < 40	1				1	
Age ≥40	1.220	0.845–1.960			**1.835**	**1.077–3.127**
Education no/elementary school	1					
Upper sec school	1.362	0.817–2.269				
University	**1.873**	**1.017–3.450**				
Empl/self–empl.	1					
Not	**0.391**	**0.259–0.590**				

Multivariate adjusted odds ratios (OR) and 95% confidence interval (CI). Crude model: univariate regression model, *n =* 406. Model I: backward regression model for functions, *n =* 345. Model II: backward adjusted OR for baseline demographics, *n =* 345. Significant OR and CI in bold face (*p =* <0.05). MMR start (IN), completed MMR (OUT). SLDP = sick leave/disability pension year before MMR.

### Factors associated with- sick leave at month four after MMR

In the crude model, different functional limitations both at MMR start and completed MMR, and also previous SLDP-episodes were associated with sick leave at month four. EQ-5D ≥ 0.5 at MMR start and at completed MMR were both associated with not being on SL month four after MMR (Table 4).

**Table 4:** Regression models for sick leave month four after MMR start

	**Crude Model**		**Model I**		**Model II**	
			**R**^**2**^ **= 0.141**		**R**^**2**^ **= 0.311**	
	**OR**	**CI**	**OR**	**CI**	**OR**	**CI**
Sensation of pain (b280) IN	1					
Moderate	**2.441**	**1.098–5.426**				
Severe/total	**3.724**	**1.688–8.214**				
Sensation of pain (b280) OUT	1					
Moderate	**2.356**	**1.344–4.131**				
Severe/total	**4.200**	**2.320–7.601**				
Problems with exercise tolerance functions (b355) IN	1					
Moderate	**2.083**	**1.273–3.410**				
Severe/total	**2.375**	**1.335–4.224**				
Problems with exercise tolerance functions (b355) OUT	1		1		1	
Moderate	**2.906**	**1.814–4.655**	**2.160**	**1.257–3.712**	**2.195**	**1.216–3.962**
Severe/total	1.653	0.828–3.298	0.748	0.324–1.728	0.979	0.397–2.415
Problems with mobility in joints function (b710) IN	1					
Moderate	1.584	0.938–2.677				
Severe/total	**2.358**	**1.306–4.255**				
Problems with mobility in joints function (b710) OUT	1		1		1	
Moderate	**2.381**	**1.429–3.966**	**2.046**	**1.151–3.637**	1.674	0.890–3.149
Severe/total	3.689	**2.000–6.805**	**3.051**	**1.502–6.200**	**2.644**	**1.206–5.795**
EQ–5D <0.5 IN	1		1		1	
≥ 0.5	**0.515**	**0.330–0.802**	0.608	0.366–1.009	0.587	0.336–1.026
EQ–5D <0.5 OUT	1					
≥ 0.5	**0.423**	**0.274–0.652**				
Sex	1					
Sex (man)	0.712	0.417–1.215				
SLDP–episode year before (no)	1				1	
One episode	**5.898**	**3.252–10.696**			**4.998**	**2.562–9.750**
> 1 episode	**8.494**	**4.604–15.683**			**7.869**	**3.894–15.900**
Married (yes)	1					
no	1.338	0.876–2.045				
Born in Sweden	1					
not	1.526	0.929–2.508				
Age < 40	1				1	
≥ 40	0.736	0.539–1.302			**0.527**	**0.298–0.932**
Education no/elementary school	1					
Upper sec school	0.965	0.548–1.698				
University	0.779	0.403–1.508				
Empl/self–empl	1					
Not	1.102	0.705–1.724				

Multivariate adjusted odds ratios (OR) and 95% confidence interval (CI). Crude model: univariate regression model, *n =* 361. Model I: backward regression model for functions, *n =* 306. Model II: backward adjusted OR for baseline demographics, *n =* 306. Significant OR and CI in bold face (*p =* <0.05). MMR start (IN), completed MMR (OUT). SLDP = sick leave/disability pension year before MMR.

In model I (R^2^ = 0.14), moderate problems with exercise tolerance functions at completed MMR, moderate and more severe problems with mobility in joints functions at completed MMR were associated with being on SL month four after MMR.

In the final, adjusted model II (R^2^ = 0.31), moderate problems with exercise tolerance functions (OR 2.2, CI 1.2–4.0, *p =* 0.009) at completed MMR, more severe problems with mobility in joints functions at completed MMR (OR 2.6, CI 1.2–5.8, *p =* 0.015), one (OR 5.0, CI 2.6–9.8, *p <* 0.000) or more than one (OR 7.9, CI 3.9–15.9, *p <* 0.000) SLDP-episode were associated with being on SL month four after MMR start. Being > 40 years was associated with not being on SL (OR 0.5, CI 0.3–0.9, *p =* 0.028) and having an EQ-5D ≥ 0.5 at MMR start was not significantly associated with not being on sick leave at month four after MMR, but with a low *p-*value (OR 0.6, CI 0.3–1.0, *p =* 0.061) (Table 4).

## Discussion

### On the results

Focus for the National rehabilitation program was to promote work ability. In this study 73.6% of the patients who were not on SLDP the year before MMR rated work ability as limited at MMR start. After MMR in this group only 34.1% reported work ability limitations. The results in this study indicate that MMR is effective also for patients not on SL and we believe that it is important to focus on patients who perceive work ability limitations but are not at SL. Often, work ability limitations are compared to being on sick leave or disability pension, but there are no perfect matches between sick leave and work ability limitations [38]. Being older than 40 years (both males and females) in our study was associated with not being on sick leave four month after MMR which was unexpected as function and HRQoL is expected to decrease in higher age. Earlier studies have indicated that older women have better quality of life [39], but population surveys indicate that EQ-5D decreases with age [32]. It is important to take into account for older persons their need for rehabilitation, since today they are expected to work at higher age and are a requested resource in society. In this study, levels of education or gender did not influence SL at month four after MMR or EQ-5D at three month follow-up but previous studies indicate that low education has an impact on RTW [40].

Function, HRQoL and work ability and sick leave were used as patient reported out-comes in this study. At MMR start, more than half of the patients had EQ-5D < 0.5. In general, EQ-5D improved during MMR, but still patients in this study had low EQ-5Ds and did not reach the level for a Swedish normal population [31,32]. The group with no SLDP the year before MMR start was at completed MMR close to a suggested cut-off value for having work ability [41]. Having an EQ-5D ≥ 0.5 at MMR start, was not significantly associated with not being on sick leave at month four after MMR start but previous studies indicate that EQ-5D can predict RTW for patients with neck/back pain, on sick leave for > 28 days [41] and we suggest that EQ-5D can be used as a predictor of future sick leave and when planning rehabilitation.

Having SLDP the year before MMR, was associated with having an EQ-5D < 0.5 at three month follow-up. In this study the group with no SLDP the year before MMR differentiate, but in an RCT, Shiri et al. [42] found that part-time sick leave at an early stage, may be advantageous to improve perceived HRQoL. SLDP-episodes the year before MMR was associated with being on sick-leave at month four after MMR start. This indicates that SLDP-history is important information, when planning for rehabilitation and sick leave and we believe that it is easy for patients to remember if they have had any previous SLDP-episodes. Earlier studies support that SLDP is a predictor for RTW [40,43].

An ICF-based structure is recommended when reporting about different health states in relation to MSD [44] but there is need for an improved operationalisation of the ICF, to obtain a more solid reliability [45]. In this study, three different functions were assessed. In general, functions improved after MMR. Functional limitations at completed MMR were associated with SL month four after MMR. This is in line with how work ability and the need for sick leave should be assessed in Sweden; not based on the diagnosis rather if there are functional limitations that affect work ability. What we do not know is how these functional limitations are related to the patient’s actual work demands [18,19]. Lydell et al. [43] has found that functional capacity, among other factors, was a predictor of long-term RTW. Planning for using assessments involving such predictors, may be beneficial in planning a sick leave period, at an early stage.

### Methodological strengths and weaknesses

A major strength in this study is that the final cohort was drawn from a cohort of all patients that were directed to MMR in Skåne County, which is a region that is representative for the whole Swedish population, in terms of demographics. Data is continuously ascertained from different PHC centres, which all used the same questionnaires and were automatically transferred to SHCR. At the time for this study, no matched controls were available.

In this study, EQ-5D was applied, which is widely used and has been found to have a good prediction of RTW for sick-listed patients with neck/back pain [41]. No Swedish tariff for EQ-5D is today available and mainly a British tariff is used [31-33] but recently, a Danish tariff has been introduced [46]. A concern would be that the UK tariffs and the potential tariffs for Swedish data differ. In 2011, a new version, offering five levels of answering alternatives, was introduced [47].

The question about self-rated work ability was answered by those who were employed/self-employed. This is especially significant in the group with full-time SLDP, where 75.4% were un-employed at MMR start. Previous studies show that self-reported work ability, give reliable information concerning patients work ability and can predict future sick leave [48,49] and a sustainable RTW [50]. We suggest that the single-item question from Work Ability Index (WAI) should be used.

## Conclusions

Both patients on SLDP or at risk for SLDP seem to benefit from MMR. We believe that it is important to offer MMR also to patients ≥ 40 years old and also focus patients that perceive work ability limitations, and are at risk for work disability, all though not yet at sick leave. Focusing EQ-5D and self-rated work ability, could contribute to create tailor-made interventions and well-defined, time- coordinated interventions may prevent future sick leave and increase HRQoL. Still, there is need to find out what components in MMR that are actually efficient.

## Competing interests

The authors declare that they have no competing interests.

## Authors’ contributions

All four authors (KS, AJ, IP, BG) designed the study, participated in data collection and data analysis. KS performed manuscript writing and the final manuscript was approved by all four authors (KS, AJ, IP, BG).

## Pre-publication history

The pre-publication history for this paper can be accessed here:

http://www.biomedcentral.com/1471-2474/14/57/prepub

## References

[B1] European Trade Union Institute (ETUI) Musculoskeletal disorders. An ill-understood pandemic 2007 Brussel: ETUI http://www.etui.org/Publications2/Guides/Musculoskeletal-disorders.-An-ill-understood-pandemic

[B2] Beavan S McGee R Quadrello T Fit for Work? Musculoskeletal Disorders and the Swedish Labour Market. The Work Foundation. Fit for work Europe 2010 http://www.fitforworkeurope.eu/default.aspx.locid-0afnew00j.Lang-EN.htm

[B3] Lidwall U Long-term Sickness Absence. Aspects of Society, Work and Family PhD-thesis 2010 Karolinska Institutet: Department of Clinical Neuroscience http://diss.kib.ki.se/2010/978-91-7409-821-1/thesis.pdf

[B4] Alexanderson K Norlund A Sickness absence- causes, consequences, and physicians’ certified sick leave practice. A systematic literature review by the Swedish council on technology assessments in health care Scand J Publ Health 2004 63 supplement 1 263 10.1080/14034950410003826 15513647

[B5] Eriksson HG von Celsing AS Wahlström R Jansson L Zander V Wallman T Sickness absence and self-reported health a population-based study of 43, 600 individuals in central Sweden BMC Publ Health 2008 8 426 10.1186/1471-2458-8-426 PMC2627845 19116000

[B6] Linton SJ A review of psychological risk factors in back and neck pain Spine 2000 25 9 1148 1156 10.1097/00007632-200005010-00017 10788861

[B7] The Swedish Social Insurance Agency (Försäkringskassan) Sjukfrånvaron i Sverige-på väg mot Europeiska nivåer? Utvecklingen i åtta länder 1990–2007 (Sick leave in Sweden- towards European levels? Development in eight countries 1990–2007). Social Insurance report 2009:10 Social Insurance report 2009:10 Stockholm [http://www.forsakringskassan.se/wps/wcm/connect/bfecf64a-e116-4704-b608-4132e90ee2c4/socialforsakringsrapport_2009_10.pdf?MOD=AJPERES]. (In Swedish)

[B8] The National Board of Health and Welfar Försäkringsmedicinskt beslutsstöd (Guide-lines for sick-listing) 2007 [http://www.socialstyrelsen.se/riktlinjer/forsakringsmedicinsktbeslutsstod]. (In Swedish)23580820

[B9] Ministry of Health and Social affairs and Swedish Association of Local Authorities and Regions (SALAR) Agreement on arrangements to decrease sick leave Dnr 2005/2453 http://www/inspsf.se/digitalAssets/0/887_2011-13-pdf

[B10] Swedish Association of Local Authorities and Regions (SALAR) Rehabiliteringsgarantin (The national rehabilitation program) 2008 [http://www.skl.se/web/Rehabiliteringsgarantin.aspx]. (In Swedish)

[B11] Swedish Government En reformerad sjukskrivningsprocess för ökad återgång i arbete (A new sick-listing process for increased return to work). Prop.2007/08:136 Swedish Government, Ministry of Health and Social Affairs [http://regeringen.se/sb/d/108/a/101584]. (In Swedish)

[B12] The Swedish Parliament Förordning om bidrag till företagshälsovård med vissa insatser inom rehabiliteringsområdet (Decree on grants to occupational health services in rehabilitation 2009). SFS 2009:1423 [http://www.lagboken.se/files/SFS/2009/091423.PDF#search=%22f%C3%B6rs%C3%A4kringskassan%20%C3%B6ka%22]. (In Swedish)23580820

[B13] The Swedish Social Insurance Inspectorate Uppföljning av sjukskrivningsmiljarden 2010 (The sick-listing billion 2010) 2011 [http://www.inspsf.se/digitalAssets/0/887_2011-13.pdf]. (In Swedish)19895499

[B14] Busch H Bodin L Bergström G Jensen IB Patterns of sickness absence a decade after pain-related multidisciplinary rehabilitation Pain 2011 152 1727 1733 10.1016/j.pain.2011.02.004 21507573

[B15] Jensen IB Busch H Bodin L Hagberg J Nygren Å Bergström G Cost-effectiveness of two rehabilitation programmes for neck and back pain patients: a seven year follow-up Pain 2009 142 202 208 10.1016/j.pain.2008.12.015 19217717

[B16] Kuoppala J Lamminpää A Rehabilitation and work ability: a systematic literature review J Rehab Med 2008 40 796 804 10.2340/16501977-0270 19242615

[B17] Williams RM Westmorland MG Lin CA Schmuck G Creen M Effectiveness of workplace rehabilitation interventions in the treatment of work-related low back pain: a systematic review Disabil Rehabil 2007 29 8 607 624 10.1080/09638280600841513 17453982

[B18] Ilmarinen J Aging workers Occup Environ Med 2001 58 546 552 10.1136/oem.58.8.546 11452053 PMC1740170

[B19] Ilmarinen J Multidimensional work ability model. Finnish Institute of Occupational Health. Helsinki. Modified 07.06.2011 2011 [http://www.ttl.fi/en/health/wai/multidimensional_work_ability_model/pages/default.aspx]

[B20] Nordenfelt L The concept of work ability 2008 Brussels: Peter Lang

[B21] Tengland PA The concept of work ability J Occup Rehabil 2011 21 2 275 285 10.1007/s10926-010-9269-x 21052807

[B22] Ståhl C Svensson T Petersson G Ekberg K The work ability divide: holistic and reductionistic approaches in Swedish interdisciplinary rehabilitation teams J Occup Rehabil 2009 19 264 273 10.1007/s10926-009-9183-2 19488838

[B23] Ståhl C Svensson T Petersson G Ekberg K Swedish rehabilitation professionals’ perspectives on work ability assessments in a changing sickness insurance system Disabil Rehabil 2011 33 15–16 1373 1382 21082871 10.3109/09638288.2010.532282

[B24] Arrelöv B Alexanderson K Hagberg J Löfgren A Nilsson G Ponzer S Dealing with sickness certification- a survey of problems and strategies among general practitioners and orthopaedic surgeons BMC Publ Health 2007 7 273 10.1186/1471-2458-7-273 PMC2089078 17910746

[B25] Löfgren A Hagberg J Arrelöw B Ponzer S Alexanderson K Frequency and nature of problems associated with sickness certification tasks: a cross-sectional questionnaire study of 5455 physicians Scand J Prim Health Care 2007 25 3 178 185 10.1080/02813430701430854 17846937 PMC3379778

[B26] Stigmar K Grahn B Ekdahl C Work ability- experiences and perceptions among physicians Disabil Rehabil 2010 32 21 1780 1789 10.3109/09638281003678309 20230251

[B27] Stigmar K Ekdahl C Grahn B Work ability- Concept and assessment from a physiotherapeutic perspective. An interview study Physiother Theory Pract 2012 28 5 344 354 10.3109/09593985.2011.622835 22087705

[B28] Fadyl JK McPherson KM Schlüter PJ Turner-Stokes L Factors contributing to work-ability for injured workers: literature review and comparison with available measures Disabil Rehabil 2010 32 14 1173 1183 10.3109/09638281003653302 20170279

[B29] Euroqol http://www.euroqol.org

[B30] Rabin R de Charro F EQ-5D: a measure of health status from the EuroQol Group Ann Med 2001 33 5 337 343 10.3109/07853890109002087 11491192

[B31] Burström K Johannesson M Diderichsen F Health-related quality of life by disease and socio-economic group in the general population in Sweden Health Policies 2001 55 51 69 10.1016/S0168-8510(00)00111-1 11137188

[B32] Burström K Johannesson M Rehnberg C Deteriorating health status in Stockholm 1998–2002: results from repeated population surveys using the EQ-5D Qual Life Res 2007 16 1547 1553 10.1007/s11136-007-9243-z 17828580

[B33] Dolan P Modelling valuations for EuroQol health states Med Care 1997 35 11 1095 1108 10.1097/00005650-199711000-00002 9366889

[B34] World Health Organisation International Classification of Functioning, Disability and Health (ICF) http://www.who.int/classifications/icf/en/

[B35] World Health Organisation International Classification of Diseases and Related Health Problems (ICD) 10 systemhttp://apps.who.int/classifications/icd10/browse/2010/en

[B36] Altman DG Practical Statistics for Medical Research 1999 USA: Chapman & Hall

[B37] Peacock J Kerry S Presenting Medical Statistics from Proposal to Publication 2010 United Kingdom: Lightning Source UK Ltd

[B38] Johansson G Hultin H Moller J Hallqvist J Kjellberg K The impact of adjustment latitude on self-assessed work ability in regard to gender and occupational type Scand J Occup Ther 2012 19 350 359 10.3109/11038128.2011.603354 21854104

[B39] Ekström H Hovelius B Quality of life and hormone therapy in women before and after menopaus Scand J Prim Health Care 2000 18 115 121 10.1080/028134300750019025 10944068

[B40] Lindell O Johansson S-E Strender L-E Predictors of stable return-to-work in non-acute, non-specific spinal pain: low total prior sick-listing, high self-prediction and young age. A two-year prospective cohort study BMC Fam Pract 2010 11 53 10.1186/1471-2296-11-53 20646286 PMC2919451

[B41] Hansson E Hansson T Jonsson R Predictors for work ability and disability in men and women with low-back or neck problems Eur Spine J 2006 15 780 793 10.1007/s00586-004-0863-5 15937677 PMC3489465

[B42] Shiri R Kausto J Martimo K-P Kaila-Kangas L Takala E-P Viikari-Juntura E Health-related effects of early part-time sick leave due to musculoskeletal disorders: a randomized controlled trial Scand J Work Environ Health 2012 Online-first-article. http://dx.doi.10.5271/sjweh.3301 10.5271/sjweh.3301 22538838

[B43] Lydell M Grahn B Månsson J Baigi A Marklund B Predictive factors of sustained return to work for persons with musculoskeletal disorders who participated in rehabilitation Work 2009 33 317 328 19759430 10.3233/WOR-2009-0879

[B44] Weigl M Cieza A Kostanjsek N Kirschneck M Stucki G The ICF comprehensively covers the spectrum of health problems encountered by health professionals in patients with musculoskeletal conditions Rheumatology 2006 45 10 1247 1254 10.1093/rheumatology/kel097 16567355

[B45] Hilfiker R Obrist S Christen G Lorenz T Cieza A The use of the comprehensive international classification of functioning, disability and health core set for low back pain in clinical practice: a reliability study Physiother Res Int 2009 14 3 147 166 10.1002/pri.436 19194959

[B46] Wittrup-Jensen KU Lauridsen J Gudex C Pedersen KM Generation of a Danish TTO value set for EQ-5D health states Scand J Publ Health 2009 37 459 466 10.1177/1403494809105287 19411320

[B47] Herdman M Gudex C Lloyd A Janssen MF Kind P Parking D Bonsel G Badia X Development and preliminary testing of the new five-level version of EQ-5D (EQ-5D-5 L) Qual Life Res 2011 20 1727 1736 10.1007/s11136-011-9903-x 21479777 PMC3220807

[B48] Ahlström L Grimby-Ekman A Hagberg M Dellve L The work ability index and single-item question: associations with sick leave, symptoms, and health- a prospective study of women on long-term sick leave Scand J Work Environ Health 2010 36 5 404 412 http://dx.doi.org/10.5271/sjweh.2917 10.5271/sjweh.2917 20372766

[B49] Alavinia SM de Boer AGEM van Duivenbooden JC Frings-Dresen MHW Burdorf A Determinants of work ability and its predictive value for disability Occup Med 2009 59 32 37 10.1093/occmed/kqn148 19073989

[B50] Kuijer PPFM Gouttebarge V Wind H van Duivenbooden C Sluiter JK Frings-Dresen MHW Prognostic value of self-reported work ability and performance-based lifting tests for sustainable return to work among construction workers Scand J Work Environ Health 2012 Online-first-article. http://dx.doiu.org/10.5271/sjweh.3302 10.5271/sjweh.3302 22538928

